# A Mediterranean Diet Mix Has Chemopreventive Effects in a Murine Model of Colorectal Cancer Modulating Apoptosis and the Gut Microbiota

**DOI:** 10.3389/fonc.2019.00140

**Published:** 2019-03-12

**Authors:** Giulia Piazzi, Anna Prossomariti, Maurizio Baldassarre, Claudio Montagna, Paola Vitaglione, Vincenzo Fogliano, Elena Biagi, Marco Candela, Patrizia Brigidi, Tiziana Balbi, Alessandra Munarini, Andrea Belluzzi, Milena Pariali, Franco Bazzoli, Luigi Ricciardiello

**Affiliations:** ^1^Department of Medical and Surgical Sciences, University of Bologna, Bologna, Italy; ^2^Center for Applied Biomedical Research (CRBA), S. Orsola-Malpighi Hospital, University of Bologna, Bologna, Italy; ^3^Department of Agricultural Sciences, University of Naples, Portici, Italy; ^4^Food Quality and Design Group, Wageningen University, Wageningen, Netherlands; ^5^Department of Pharmacy and Biotechnology, University of Bologna, Bologna, Italy; ^6^Pathology Unit, S. Orsola-Malpighi Hospital, University of Bologna, Bologna, Italy; ^7^Gastroenterology Unit, S. Orsola-Malpighi Hospital, University of Bologna, Bologna, Italy

**Keywords:** colorectal cancer, chemoprevention, microbiota, Mediterranean diet, omega-3

## Abstract

**Objectives:** Unhealthy dietary patterns have been associated with colorectal cancer (CRC) onset while Mediterranean Diet (MD) has been proposed for CRC prevention. This study evaluated the effect of a Mediterranean Diet Mix (MD-MIX) on colonic tumors development in A/J mice fed a low-fat (LFD) or a high-fat western diet (HFWD), and injected with the procarcinogen azoxymethane (AOM).

**Materials and Methods:** Forty A/J male mice were randomly assigned into four feeding arms (10 mice/arm; LFD, LFD-MD-MIX, HFWD, HFWD-MD-MIX) to be treated with AOM. Ten mice were exposed to the diets alone (Healthy LFD and Healthy HFWD) to be used as control. Tumor incidence and multiplicity were evaluated at sacrifice. Mucosal fatty acid content and urinary phenolic compounds were assayed by mass spectrometry. Apoptosis was evaluated by TUNEL assay and gene expression markers. Cell proliferation was evaluated by Ki67 immunohistochemistry. Microbiota composition was assessed at different time points by 16S RNA sequencing.

**Results:** A tumor incidence of 100% was obtained in AOM-treated mice. The MD-MIX supplementation was able to reduce the number of colonic lesions in both LFD and HFWD-fed mice and to induce apoptosis, in particular in the LFD-MD-MIX arm. Moreover, a preventive effect on low-grade dysplasia and macroscopical lesions (>1 mm) development was found in HFWD-fed mice together with a regulation of the AOM-driven intestinal dysbiosis.

**Conclusions:** MD-MIX was able to counteract CRC development in mice under different dietary backgrounds through the regulation of apoptosis and gut microbiota.

## Introduction

Colorectal cancer (CRC) remains one of the most frequently diagnosed malignancies worldwide with 1.8 million new cases estimated in 2018 (1). Globally, CRC incidence greatly diverge among different geographic areas reaching the highest rate of incidence in developed countries where a high-fat western dietary pattern is largely adopted (2). In particular, the highest CRC incidence rates are found in Australia/New Zealand, Northern America, Eastern Asia and some parts of Europe (e.g., Hungary and Norway) (1).

A clear relationship between long-term unhealthy dietary habits and increased risk of developing CRC is widely recognized (3), while prudent dietary patterns have been associated to CRC prevention (4). Among healthy dietary patterns, a pescovegetarian diet has been correlated with an overall lower CRC incidence highlighting the importance of fruits, vegetables, cereals, and fish consumption for CRC risk reduction (5). Moreover, it is known that Mediterranean Diet (MD), which is characterized by a daily consumption of whole grains, legumes, nuts, olive oil, fish, fruits, and vegetables protects against colonic adenoma recurrence and CRC risk in different cohort studies (6–8). Importantly, data obtained from the European Prospective Investigation into Cancer and nutrition (EPIC) study involving 10 European countries and about 520,000 healthy subjects (aged 25–70 years) showed that adherence to Mediterranean Diet was associated with a reduction of CRC risk (8–11%) (9). The preventive effects of these prudent dietary patterns on CRC are mainly associated with the high content of polyphenols and ω-3 polyunsaturated fatty acids (ω-3-PUFAs) (10–12). Some of the phenolic compounds commonly found in the MD are: catechins (from apples and walnuts), ferulic acid (from whole grain), naringenin (from tomatoes), and hydroxytyrosol (from olive oil) (13). We previously demonstrated that polyphenols extracted from annurca apple as well as the ω-3-PUFA Eicosapentaenoic acid as free fatty acid (EPA-FFA) were able to exert chemopreventive properties in the Apc^Min/+^ mouse model of familial adenomatous polyposis (14, 15). Also, the anticancer effect of walnut consumption have been recently demonstrated in the Azoxymethane (AOM) mouse model of CRC (16).

There is mounting evidence that the gut microbiota composition is critically influenced by dietary habits and that an imbalance in the relative abundance of specific bacterial strains may have a pivotal role in CRC initiation and progression (17). Recently, the impact of MD on the gut microbiota structure has been investigated in humans demonstrating that the beneficial effects associated with MD may be driven by changes in the gut microbiota composition (18, 19).

Although the anticarcinogenic properties of single food-derived compounds have been evaluated in CRC (20), the combined effect of food bioactives, typically consumed in the MD regimen, has not been investigated so far.

In this study, we hypothesized that extracts from MD components in combination with the fish-derived EPA-FFA (referred as MD-MIX) may be able to counteract the development of CRC in AOM-treated mice undergoing different dietary protocols. We found that the MD-MIX prevented the development of colonic malignant lesions mainly acting on apoptosis in mice fed a low-fat diet (LFD) or modulating the gut microbiota structure in mice fed a high-fat western diet (HFWD).

## Materials and Methods

### Mediterranean Diet Mix Composition

The Mediterranean Diet Mix (MD-MIX) was obtained by combining the ω-3 PUFA EPA-FFA (provided in the diet as outlined in the next paragraph) and a mix of bioactive phytochemicals extracts resembling the main categories constituting the Mediterranean diet and contributing to the total phytochemicals intake (Annurca apples 25%, cherry tomatoes 25%, walnuts 15%, wholegrain wheat 15%, and olive oil 20%). Phytochemicals sources and quantity were selected in order to have a good balance of the different families of polyphenols (flavanols, anthocyanins, ellagitannins, secoiridoids).

The polyphenol profile of the mix of extracts was characterized (see Supplementary Methods) and its concentration was expressed as equivalents of the most represented polyphenols: catechins (from apples and walnuts), ferulic acid (from wholegrain wheat), naringenin (from tomatoes), and hydroxytyrosol (from olive oil) (Supplementary Table 1). To preserve polyphenols stability a stock solution (1 mmol/L; 80:20 water: ethanol) was prepared, aliquoted and stored at −80°C until use.

### Animals and Diets

Animal experiments were performed in accordance with the guidelines and regulations of the University of Bologna Animal Welfare Committee. The research protocol was approved by the Italian Institute of Health (approval number 203/2016-PR).

Fifty A/J male mice (5 weeks old) were purchased from Jackson Lab (Sacramento, CA, USA) and housed in individually ventilated cages (five mice/cage) in a temperature and humidity controlled animal facility with a 12 h light/dark cycle.

**Figure 1:**
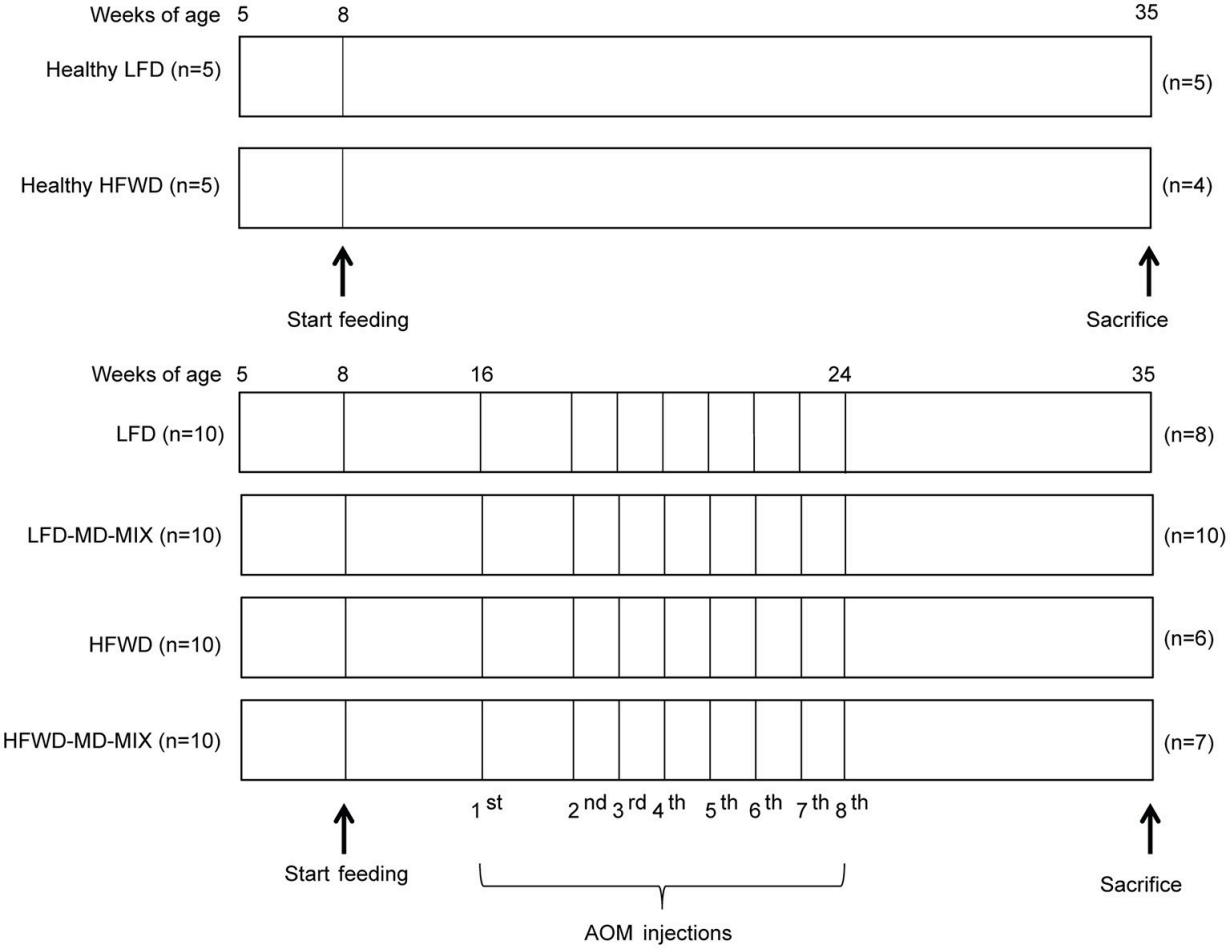
Feeding and carcinogenesis protocol. After acclimation (age 8 weeks) feeding protocols were started and maintained for 27 weeks. From week 16 LFD, LFD-MD-MIX, HFWD, and HFWD-MD-MIX mice were subjected to eight intraperitoneal AOM injections. The number of mice “n” in each arm is shown.

After a short period of acclimatization (1 week), mice were randomly assigned to the following feeding protocols (Figure 1): (1) low-fat diet (LFD: 12% calories from fat; normal calcium, cellulose, and vitamin D3 content; 3.75 kcal/g) and drinking water (Healthy LFD; *n* = 5); (2) high-fat western diet (HFWD: 45% calories from fat; low calcium, cellulose, vitamin D3 content; 4.98 kcal/g), and drinking water (Healthy HFWD; *n* = 5); (3) LFD and drinking water containing the extracts vehicle ethanol (LFD *n* = 10); (4) LFD in which soybean oil was substituted for 1% highly-purified EPA-FFA (ALFA, SLA Pharma AG, Switzerland), and drinking the mix of extracts (LFD-MD-MIX *n* = 10); (5) HFWD and drinking water containing the extracts vehicle ethanol (HFWD *n* = 10); (6) HFWD in which soybean oil was substituted for 1% highly-purified EPA-FFA and drinking the mix of extracts (HFWD-MD-MIX *n* = 10). Diets were formulated by Charles River (Lecco, Italy; see Supplementary Table 2). The beverage containing the mix of extracts was freshly prepared every day by diluting the stock solution 1:100 in water (final concentration: 10 μmol/L polyphenol equivalents). The final ethanol concentration was 0.2%. For carcinogenesis induction, LFD, LFD-MD-MIX, HFWD, and HFWD-MD-MIX arms (16-week old mice) received 8-weekly intraperitoneal injections of AOM (#A5486; Sigma-Aldrich; St. Louis, MO) under mild sedation (Figure 1). The same AOM lot (#SLBN5975V) was used for the entire experiment. Moreover, to guarantee its stability, the reagent was aliquoted and stored at −80°C until use. The first AOM injection was performed at 10 mg/kg, while all the following were performed at 7.5 mg/kg in order to reduce the risk of mortality observed after the first injection. Healthy LFD and Healthy HFWD arms did not receive AOM injections.

Body weight was monitored weekly, while rectal bleeding and diarrhea daily. Nineteen weeks after the first AOM injection, mice were euthanized through isofluorane overdose followed by cardiac puncture. Urine was collected at sacrifice by bladder aspiration and stored at −80°C. The colon was immediately removed, opened longitudinally, washed with ice-cold phosphate buffered saline, and its length was recorded. The number, location and size of macroscopical lesions was determined using a stereomicroscope and measured using a digital caliper. Fresh tissue samples from normal colonic mucosa (1 cm length) were collected from middle colon and stored at −80°C for molecular analyses or formalin-fixed and paraffin-embedded (FFPE) for immunohistochemistry (IHC). Then, the remaining colon was fixed in Bouin solution and paraffin-embedded to be processed for histopathology (21).

### Histopathology

Histopathological evaluation was performed by one expert blinded pathologist (TB) on multiple hematoxylin and eosin stained sections obtained from the whole colon of each mouse. Dysplastic areas were classified as low-grade dysplasia (LGD), high-grade dysplasia (HGD), or adenocarcinoma (AdenoK).

### Mucosal Fatty Acid Analysis

Mucosal fatty acid content was determined by gas chromatography-mass spectrometry (GC-MS) on fresh tissue samples as previously described (22).

### Determination of Urinary Polyphenols

Polyphenols were extracted from urine samples as previously reported (23) with minor modifications (see Supplementary Methods). The molecular formula and the selected ion for quantified polyphenols are reported in Supplementary Table 3.

### Immunohistochemistry and TUNEL Assay

For IHC, FFPE colonic tissues were sectioned at 4 μm thickness and processed as previously reported, using Ki67 primary antibody (1:400 Cell Signaling Technology #9027). The percentage of Ki67^+^ cells was obtained dividing the number of positive nuclei on total nuclei on 15 colonic crypts (when possible longitudinal crypts were selected). TUNEL assay was performed using DeadEnd™ Fluorometric TUNEL System (Promega, Madison, WI, USA) on FFPE normal colonic tissues. For quantitative analysis of TUNEL, ImageJ software (https://imagej.nih.gov/ij/), coupled with the Color Deconvolution plug-in, was used to quantify the percentage of positively stained nuclear area as previously (24).

### RNA Extraction and Quantitative Real-Time PCR

Total RNA was extracted from fresh normal colonic tissues using TRIzol reagent (Invitrogen™, Thermo Fisher Scientific) and RNA concentration was measured using the Nanodrop 1,000 spectrophotometer (Thermo Fisher Scientific). Then, total RNA was reverse transcribed using the High-Capacity RNA-to-cDNA™ Kit (Applied Biosystems™, Thermo Fisher Scientific) according to the manufacturer's instructions. Quantitative real-time PCR (q-PCR) reactions were performed on ICycler thermal cycler (Biorad, Hercules, USA) using the SYBR®Select Master Mix for CFX and PrimeTime qPCR Primers for *Bax* (Mm.PT.58.14012210) and *Bcl2* (Mm.PT.58.7362966) (Integrated DNA Technologies, Coralville, USA) genes. *Gapdh* was used as reference gene for mRNA normalization. Fold induction levels were obtained using the 2^−ΔΔ*Ct*^ method by normalizing against the reference gene. Data were plotted as *Bax*/*Bcl2* ratio.

### Microbiota Analysis and Bioinformatics

For microbiota analysis fecal samples were collected from mice at different time points: 9 (T1: 1 week after the beginning of the feeding protocol), 17 (T2: 1 week after the first AOM injection), and 35 (T3: at the sacrifice) weeks of age. Feces from mice grouped in the same cage were pooled at each time point and total bacterial DNA was extracted using the QIAmp DNA Stool Mini Kit (Qiagen, Hilden, Germany). Microbiota data were obtained by sequencing the V3-V4 region of the 16S rRNA gene by using an Illumina platform. For each sample, the V3-V4 region of the 16S rRNA gene was PCR amplified using primers carrying Illumina overhang adapter sequences (25). Amplicons were purified with a magnetic bead-based clean-up system (Agencourt AMPure XP; Beckman Coulter, CA). Indexed libraries were prepared by limited-cycle PCR using Nextera technology and cleaned up with AMPure XP magnetic beads. Libraries were pooled at equimolar concentrations (4 nM), denatured and diluted to 6 pM before loading onto the MiSeq flow cell. A 2 × 300 bp paired end protocol was used, according to the manufacturer's instructions (Illumina, San Diego, CA). Raw sequences were processed using a pipeline combining PANDAseq and QIIME (26, 27). High-quality reads, as selected using the default values in QIIME, were binned into operational taxonomic units (OTUs) according to the taxonomic threshold of 97% using UCLUST (28), through an open-reference strategy. Taxonomy was assigned using the RDP classifier against Greengenes database (May 2013 release). The percentages of OTUs assigned to the different bacterial phylogenetic groups (from genus to phylum) were calculated as relative abundances for all samples. Average abundances were calculated for each time point and for all diet combinations. Values for bacterial genera present at a relative abundance >0.05% in at least one sample were used to produce a heatmap with horizontal scaling, plotting the z score (i.e., number of standard deviations from the mean, from −3 to 3) for each time point. The heatmap was generated using the function heatplot within the R package MADE4.

Beta diversity was estimated by computing weighted UniFrac distances, which were used for Principal Coordinates Analysis (PCoA) performed using vegan package in R statistical software.

### Statistical Analysis

Statistical analysis was performed using Graphpad 6.0 Software (GraphPad Software Inc., CA, USA). The mean differences among groups were calculated using one-way two-tailed ANOVA followed by Tukey's multiple comparison *post-hoc* test. *P* < 0.05 were considered statistically significant.

## Results

### MD-MIX Counteracts CRC Development

**Figure 2:**
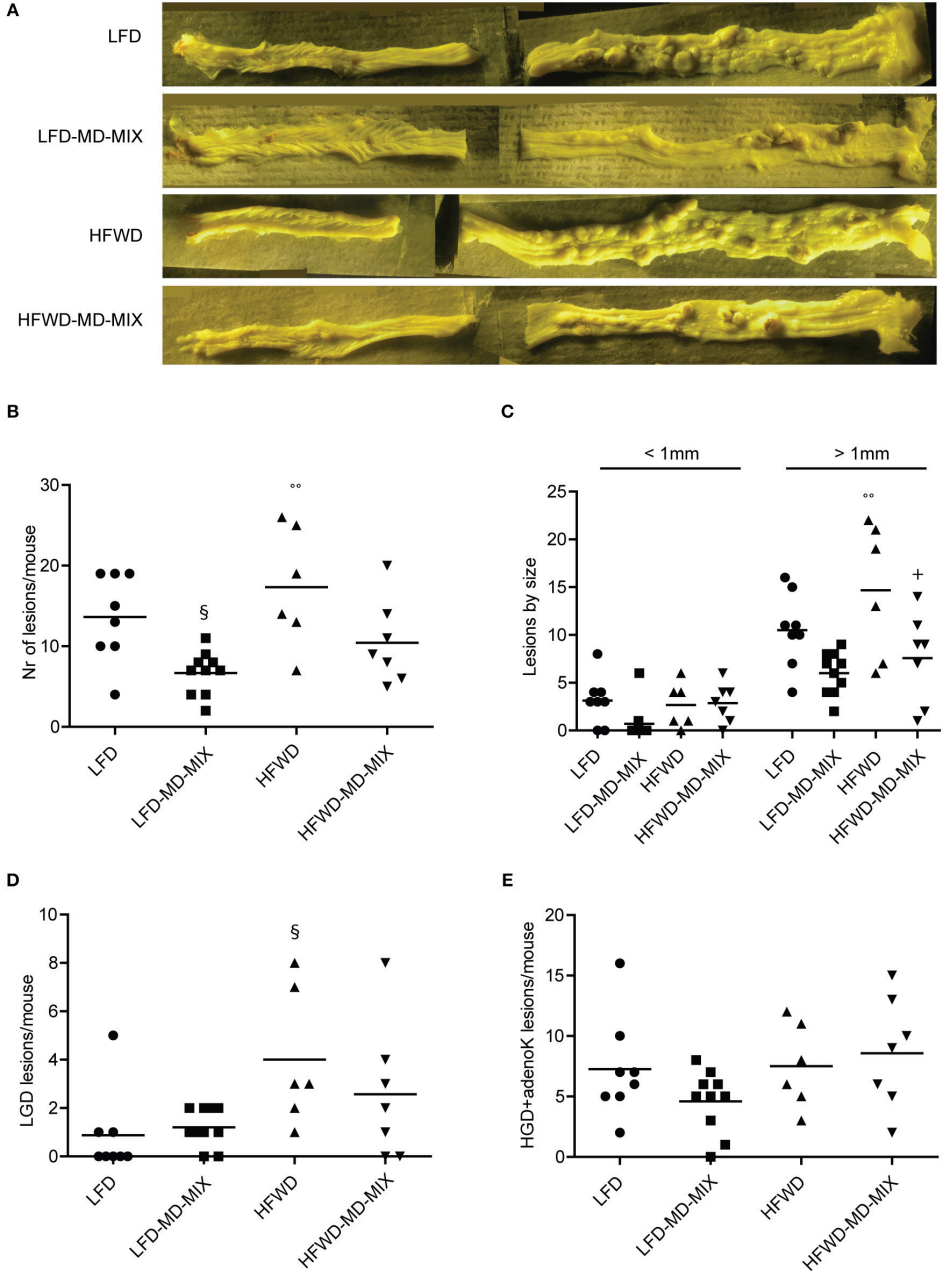
Effect of MD-MIX on tumor multiplicity, size and histological distribution of colonic lesions. **(A)** Representative images of colons fixed in Bouin's solution; **(B)** Tumor multiplicity (number of macroscopic lesions/mouse) (ANOVA *p* = 0.0027); **(C)** Number of macroscopic colonic lesions by size (ANOVA *p* = 0.0052 for lesions >1 mm); **(D)** Number of low grade dysplasia (LGD) lesions/mouse (ANOVA *p* = 0.0329); **(E)** Number of high grade dysplasia+ adenocarcinoma lesions (HGD+adenoK)/mouse. Data are shown as individual values. *n* = 8 LFD; *n* = 10 LFD-MD-MIX; *n* = 6 HFWD; *n* = 7 HFWD-MD-MIX. *P*-values were determined using one-way two-tailed ANOVA followed by Tukey's multiple comparison *post-hoc* test. ^§^*p* < 0.05 vs. LFD; ^°°^*p* < 0.01 vs. LFD-MD-MIX; ^+^*p* < 0.05 vs. HFWD.

The primary endpoint of this study was to evaluate the chemopreventive effect of the MD-MIX on colon carcinogenesis in AOM-injected A/J mice fed a LFD or HFWD. The A/J strain was chosen given its high sensitivity to AOM-driven colon carcinogenesis (29). Tumor incidence was 100% in AOM-exposed mice, while no tumors were observed in both LFD and HFWD healthy mice indicating that HFWD alone is not sufficient to induce tumor development during a lifespan of 27 weeks. Representative images of colons at sacrifice are reported in Figure 2A. Importantly, MD-MIX feeding significantly reduced tumor multiplicity in the LFD-MD-MIX group respect to the LFD arm and also protected against colonic lesions development in HFWD-fed mice (Figure 2B). Noteworthy, HFWD group showed a significant higher number of lesions compared to LFD-MD-MIX arm (Figure 2B). Analyzing the effect of the diet (LFD or HFWD) or MIX on the number of lesions, we found that the MIX significantly affects the number of lesions in mice fed both diets with no interaction between diet and MIX. Regarding lesion size, HFWD arm displayed a significant higher number of lesions larger than 1 mm compared to LFD and LFD-MD-MIX arms (Figure 2C). Treatment with the MD-MIX in HFWD-fed mice was associated with a significant lower number of lesions larger than 1 mm while no significant differences in terms of lesions smaller than 1 mm were observed among all groups (Figure 2C). Interestingly, histological analysis showed a higher number of LGD lesions in the HFWD arm, which was partially counteracted by the MD-MIX (Figure 2D). No significant changes were observed in the advanced lesions (high grade dysplasia and adenocarcinoma) among all groups (Figure 2E).

Colon length, which provides an indication of the severity of colonic inflammation (30), was significantly reduced upon AOM treatment in both LFD and HFWD groups compared with healthy LFD mice. Noteworthy, the exposure to the MD-MIX partially counteracted colon length shortening induced by the AOM treatment (Supplementary Figure 1).

Taken together our data demonstrate a protective effect of the MD-MIX against AOM-driven colon carcinogenesis.

### Effect of Dietary Protocols on Body Weight, Colonic EPA Incorporation and Urinary Polyphenol Content

We did not observe a significant weight increase in mice fed a HFWD compared with LFD-fed mice (Supplementary Figure 2). Moreover, AOM exposition was associated with a significant weight reduction in all treated mice independently from the diet (Supplementary Figure 2).

**Figure 3:**
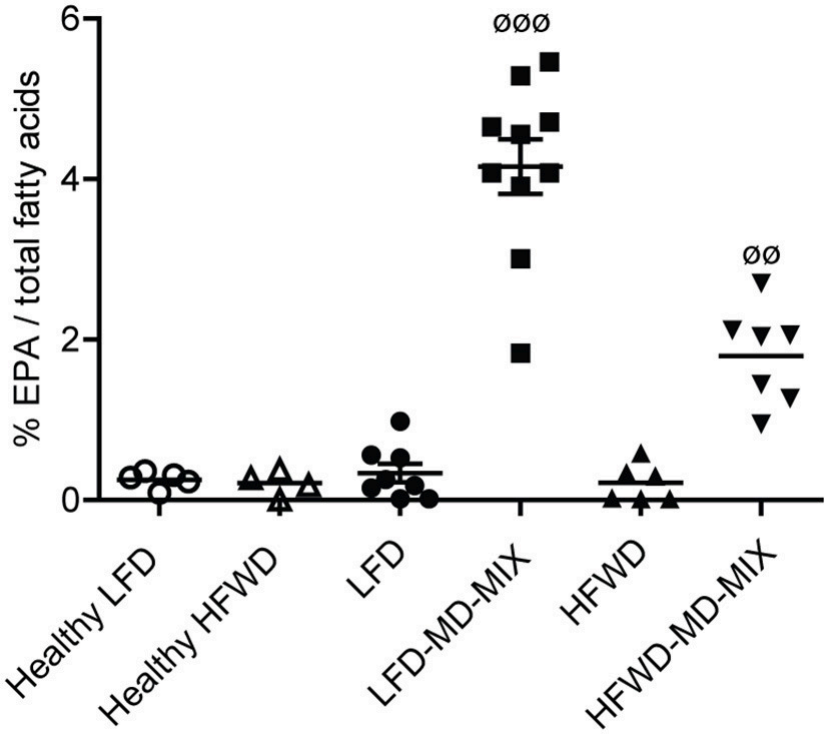
EPA incorporation on colonic tissues. Eicosapentaenoic acid (EPA; C20:5 n-3) levels in colonic tissues expressed as percentage of total fatty acids (ANOVA *p* < 0.0001). Data are shown as individual values. *n* = 5 Healthy LFD; *n* = 4 Healthy HFWD; *n* = 8 LFD; *n* = 10 LFD-MD-MIX; *n* = 6 HFWD; *n* = 7 HFWD-MD-MIX. *P*-values were obtained using one-way two-tailed ANOVA followed by Tukey's multiple comparison *post-hoc* test.^øø^*p* < 0.01 HFWD-MD-MIX vs. all groups;^øøø^*p* < 0.001 LFD-MD-MIX vs. all groups.

Colonic EPA incorporation into cellular membranes and urinary polyphenols were measured to evaluate the bioavailability of EPA and polyphenols in MD-MIX treated mice and to investigate the effect of diets (LFD vs. HFWD) and colonic carcinogenesis (AOM treated vs. healthy mice) on their metabolism. As shown in Figure 3, LFD-MD-MIX and HFWD-MD-MIX arms displayed a significant increase in the percentage content of EPA in colonic cell membranes compared to all other arms. Noteworthy, EPA levels in LFD-MD-MIX group were also significantly higher than those in HFWD-MD-MIX arm.

Fourteen polyphenols were retrieved in urine samples (Supplementary Table 4). Unexpectedly, HFWD, but not LFD feeding, was associated with an increased urinary polyphenol content which is detectable both in healthy and AOM-treated mice. Moreover, MD-MIX exposure led to a further increase in urinary polyphenol concentration in HFWD mice, but not in LFD arm (Supplementary Table 4).

These results indicate that EPA was efficiently incorporated in colonic epithelial membranes upon EPA-FFA supplementation in particular in LFD-fed mice and that urinary polyphenols excretion was increased under HFWD regimen.

### Effect of MD-MIX on Apoptosis and Cell Proliferation

**Figure 4:**
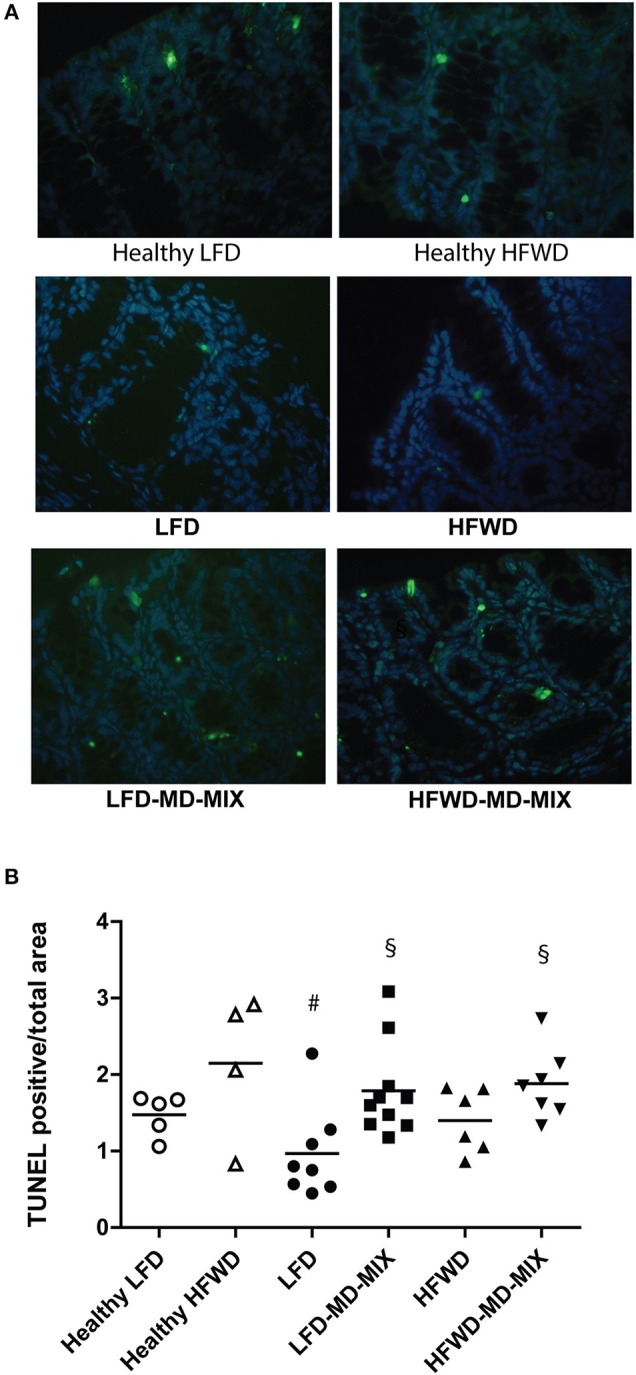
MD-MIX effects on cell apoptosis. **(A)** TUNEL representative images of colon from LFD and HFWD-fed mice (left and right column, respectively; magnification 40 X). **(B)** MD-MIX increased apoptosis (ANOVA *p* = 0.0122). Data are plotted as individual values. *n* = 5 Healthy LFD; *n* = 4 Healthy HFWD; *n* = 8 LFD; *n* = 10 LFD-MD-MIX; *n* = 6 HFWD; *n* = 7 HFWD-MD-MIX. Statistical significance was obtained using one-way two-tailed ANOVA followed by Tukey's multiple comparison *post-hoc* test. ^#^*p* < 0.05 vs. Healthy HFWD; ^§^*p* < 0.05 vs. LFD.

We then evaluated whether the MD-MIX could modulate apoptosis and cell proliferation. TUNEL staining demonstrated an induction of apoptosis in both dietary regimens treated with MD-MIX, although a stronger effect was observed in LFD-MD-MIX arm (Figures 4A,B). These data were partially confirmed by *Bax*/B*cl2* ratio showing an increase in LFD-MD-MIX arm (Supplementary Figure 3). No significant differences in Ki67 staining were observed among all groups (Supplementary Figure 4).

These results suggest that the MD-MIX may exert antineoplastic effects through a modulation of apoptosis.

### Effect of AOM and MD-MIX on Gut Microbiota Structure

The pivotal role of the diet and colon carcinogenesis in modulating the gut microbiota composition has been described (31, 32). Thus, we evaluated whether AOM treatment under different dietary regimens may induce changes in specific bacterial populations. The 16S rRNA amplicons obtained from DNA samples extracted from pooled mice feces were sequenced, resulting in 226,372 high quality sequences, ranging between 2,166 and 5,802 with an average value of 4,527 sequences per sample. Reads were clustered into 7,791 operational taxonomic units (OTUs) based on 97% similarity.

**Figure 5:**
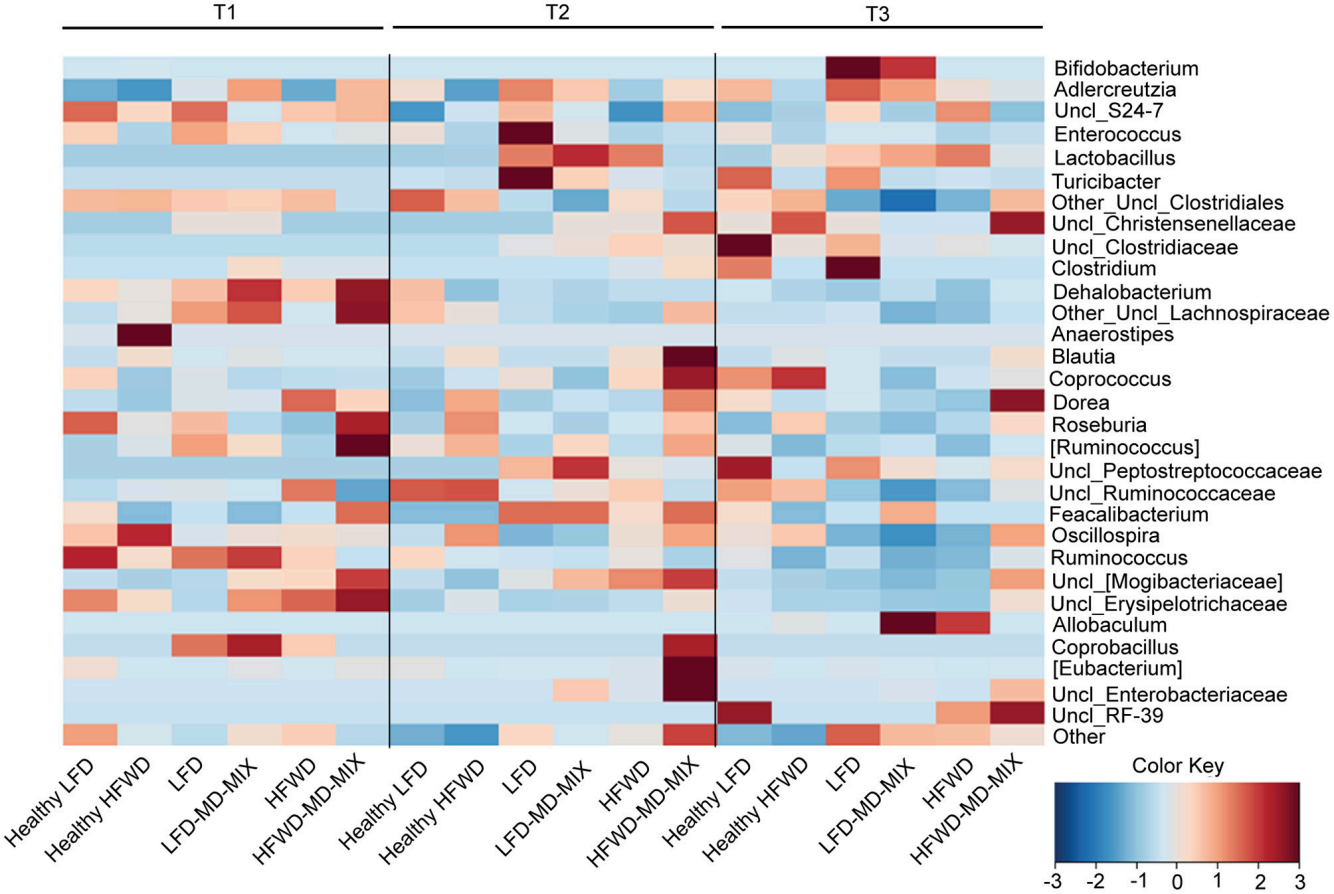
Impact of diets, AOM-treatment and MD-MIX on gut microbiota composition. Genus level average fecal microbiota profiles at time points T1, T2, and T3 for all combinations of diet (LFD and HFWD) and supplementation (MD-MIX), expressed as heatmap with horizontal scaling. Bacterial genera present at a relative abundance >0.05% in at least one sample are plotted. The color scale from blue to brown indicates the z score (i.e., number of standard deviations from the mean, from −3 to 3) for each time point. The heatmap has been generated using the function heatplot within the R package MADE4.

AOM exposure caused a gut microbiota imbalance in both LFD and HFWD-fed mice (Figure 5). Indeed, we found an early increase in Lactobacillaceae members (in particular the genus *Lactobacillus*) at T2 in both LFD and HFWD arms (from 0 to 22% of the entire ecosystem in average for both diets), which is mostly maintained at T3 (14 and 22% for LFD and HFWD, respectively). Noteworthy, LFD fed mice exposed to AOM supplementation with MD-MIX caused a further increase of Lactobacillaceae (T2: 22% LFD vs. 30% LFD-MD-MIX; T3: 14% LFD vs. 18% LFD-MD-MIX), while this effect was counteracted by MD-MIX treatment in HFWD-fed mice (T2: 22% HFWD vs. 2% HFWD-MD-MIX; T3: 22% HFWD vs. 6% HFWD-MD-MIX). Members of the Firmicutes (in particular the genus *Oscillospira*) followed an inverse pattern respect to Lactobacillaceae. Indeed, a decrease in the genus *Oscillospira* was observed in LFD, LFD-MD-MIX, and HFWD arms from T1 (8.3% in average) to T3 (2.12% in average) whereas it was increased in the HFWD-MD-MIX group (from 8.3 to 12.7%). Moreover, we detected an increase in the relative abundance of Erysipelotrichaceae (in particular the genus *Allobaculum*) in both LFD-MD-MIX (from 4.9% at T1 to 33% at T3) and HFWD arms (from 5.7% at T1 to 21.3% at T3), while a reduction was observed in HFWD-MD-MIX group (from 8.03% at T1 at 2.65% at T3). Interestingly, the MD-MIX seems to counteract the AOM dependent microbiome unbalances only upon the HFWD regimen. PCoA, based on weighted Unifrac distances among gut microbiota profiles at OTUs level, confirmed this trend in HFWD-fed mice. In fact, PCoA showed that fecal samples at all three time-points (T1, T2, and T3) from mice supplemented with the MD-MIX plotted together in the left side independently from the AOM injection. On the contrary, samples from HFWD-fed mice taken at T2 and T3 showed a progressive drift toward the right side of the plot and far from the area in which the samples from healthy mice resided (Supplementary Figure 5).

Overall, our data suggests that the impact of MD-MIX on the AOM-associated gut microbiota dysbiosis is strictly dietary dependent.

## Discussion

In this study, we aimed at testing the potential chemopreventive effects of a polyphenol extract obtained from MD components, in combination with EPA-FFA (MD-MIX), in mice subjected to AOM injections and different diets.

Importantly, we found a reduction in tumor multiplicity in mice exposed to AOM and treated with the MD-MIX. In particular, our results showed that dietary supplementation with the MD-MIX may provide protection against early carcinogenic events, also in mice exposed to HFWD.

The impact of dietary habits on CRC risk has been extensively investigated. In particular, there is a great interest in identifying an effective dietary regimen that could counteract the adverse events associated with long-term unhealthy dietary habits and the widespread development of obesity, including CRC. However, many observational human studies conducted to evaluate how food habits may affect obesity and CRC risk often suffer from confounding factors thus providing inconsistent results (32). The MD is recognized as a healthy dietary pattern. The effect of MD on CRC risk has been extensively discussed in a recent review (8). Overall, a protective effect of MD pattern on CRC risk has emerged from case-control studies and meta-analyses, although data obtained from cohort studies were less consistent. These discrepancies appeared to be mainly due to different factors including: indexes used to quantify the adherence to MD, age, sex, and tumor site. Importantly MD was found to be more protective against the development of distal CRC in young adults, particularly inhabiting the MD geographic area (8).

To the best of our knowledge this is the first study aiming to approximate a MD regimen in mice and to clarify its impact on CRC onset. Despite being less obesity-prone compared to other mice strains (i.e., C57BL/6J), we opted to use the A/J strain for its increased susceptibility to AOM-induced colon carcinogenesis (29). Indeed we obtained a 100% tumor incidence in AOM-treated mice, while no tumors were observed in healthy mice fed with HFWD alone. These results are consistent with previous literature data showing that a long-term exposure to high-fat diet is necessary to obtain colonic malignant lesions in the absence of a mutagenic agent (33).

Being chemoprevention the main objective of this study, all the analyses performed to evaluate the molecular mechanisms that precede cancer development in this setting, were conducted on non-neoplastic colonic mucosa.

In order to evaluate EPA and polyphenols bioavailability, EPA incorporation in colonic tissues and urinary excretion of polyphenols were assessed. Data showed that mice treated with the MD-MIX efficiently incorporated EPA in colon tissues, independently from the diet, although LFD-MD-MIX group showed a higher EPA percentage respect to HFWD-MD-MIX arm. Thus, it is possible that HFWD, containing a higher percentage of linoleic acid compared with LFD may result in a lower EPA incorporation. Importantly, we found a higher level of apoptosis in the MD-MIX treated mice, particularly in LFD-MD-MIX. Different dietary components present in the MD extract have been associated with apoptosis induction (34). Among these, omega-3 are known to have pro-apoptotic properties in CRC (15, 22, 35). In addition, we observed a higher polyphenol bioavailability in HFWD than LFD arms exposed to the MD-MIX. Since it is known that insoluble dietary fibers can speed intestinal transit time and bind polyphenols in the intestine (36), we hypothesized that the higher content of cellulose in LFD compared with HFWD might affect polyphenols absorption.

The role of the gut microbiota in the pathogenesis of CRC is widely demonstrated (37, 38), and it is known that dietary habits may cause relevant differences in the gut microbiota structure (39). Recently, the beneficial effect of the consumption of foods typical of a MD regimen on gut microbiota composition have been highlighted in an Italian (18) and a Spanish cohort (40).

Interestingly, in our study, despite the chemopreventive effects of MD-MIX in both LFD and HFWD arms, we observed that the MD-MIX effect in counteracting the AOM-associated gut microbiota dysbioses was strictly dietary dependent. This leads us to hypothesize that the impact of MD-MIX on the gut microbiota may act with distinct mechanisms based on dietary regimens, as previously suggested (41). Indeed, although AOM exposure led to a rapid increase in the relative abundance of Lactobacillaceae family in both LFD and HFWD arms, MD-MIX was able to redress this dysbiotic drift only in mice fed HFWD. The increase in Lactobacillaceae observed in AOM-treated mice happened concomitantly to a decrease in members of the Firmicutes phylum (mainly *Oscillospira* and *Uncl_Clostridiales*). Importantly, HFWD-MD-MIX group showed Firmicutes profile similar to healthy HFWD mice with increased abundance of *Oscillospira* genus belonging to the Ruminococcaceae family. Recent findings in a Spanish cohort living in the Mediterranean area showed an inverse association among *Oscillospira* abundance with BMI and dietary intake of proteins and cholesterol indicating that changes in specific bacterial strains may reflect differences in nutrient consumption (40). Moreover, increased levels of *Oscillospira* have been also associated with MD consumption in non-human primates respect to Western-diet consumers (42).

An overgrowth of the *Allobaculum* genera, a member of Erysipelothricaceae, was found in HFWD arm at T3. A hyperproliferation of *Allobaculum*-like bacteria has been observed in fecal samples of rats treated with the carcinogen 1,2-dimethyl hydrazine which is a metabolic precursor of AOM (43). In addition, increased levels of Erysipelotrichaceae, in particular genera *Allobaculum* in mice fed a low-fat diet have been reported (41, 44), while controversial results regarding changes in members of Erysipelotrichi class of bacteria in mice undergoing a high-fat diet or western dietary pattern have been described (45, 46). As observed for *Lactobacillus* and *Oscillospira*, the HFWD-MD-MIX maintained a healthy-like abundance of *Erysipelotrichi* as well. Interestingly, in the context of the LFD regimen, the MD-MIX treatment strengthens the increase in *Erysipelotrichi*, consolidating the dysbiotic potential of AOM.

Our data indicate that the effects of MD-MIX on the AOM-driven gut microbiota dysbiosis are strictly dependent on the underlying dietary regimen. Indeed, while MD-MIX keeps the gut microbiome ecosystem in an eubiotic configuration under HFWD exposure, it consolidates an AOM dependent dysbiotic gut microbiota signature in the LFD fed groups.

In conclusion, our findings demonstrated that the supplementation with bioactives typical of MD could be effective as a chemopreventive approach in subjects who adhere to either a balanced or a western dietary regimens. Further clinical studies involving human subjects are warranted to clarify the role of MD on CRC prevention and to investigate the potential combined effect of dietary supplementation in association with a pharmacological strategy to obtain a stronger protection.

## Data Availability

All datasets generated for this study are included in the manuscript and/or the supplementary files.

## Ethics Statement

This study was carried out in accordance with the recommendations of University of Bologna Animal Welfare Committee. The protocol was approved by the Italian Institute of Health (203/2016-PR).

## Author Contributions

GP and AP: conducting experiments, drafting of the manuscript, acquisition of data, analysis, and interpretation of the data, statistical analysis. MB and CM: technical support and acquisition of data. PV, VF, EB, MC, and PB: technical support, acquisition of data, analysis and interpretation of data. TB: analysis and interpretation of data. AM and AB: technical support, acquisition of data. MP: technical support. FB: critical revision of the manuscript for important intellectual content. LR: study concept and design, study supervision, analysis and interpretation of data, obtained funding.

### Conflict of Interest Statement

The authors declare that the research was conducted in the absence of any commercial or financial relationships that could be construed as a potential conflict of interest.
