# Supplementary material for: A Mediterranean Diet Mix Has Chemopreventive Effects in a Murine Model of Colorectal Cancer Modulating Apoptosis and the Gut Microbiota
Source: Front Oncol. 2019 Mar 12;9:140. doi: 10.3389/fonc.2019.00140 (PMC6423077; doi:10.3389/fonc.2019.00140)

A Mediterranean Diet mix has chemopreventive effects in a murine model of colorectal cancer modulating apoptosis and the gut microbiota

Giulia Piazzi[1,2#], Anna Prossomariti[1,2#], Maurizio Baldassarre[1,2], Claudio Montagna[1,2], Paola Vitaglione[3], Vincenzo Fogliano[4], Elena Biagi[5], Marco Candela[5], Patrizia Brigidi[5], Tiziana Balbi[6], Alessandra Munarini[1,2], Andrea Belluzzi[7], Milena Pariali[2], Franco Bazzoli[1], Luigi Ricciardiello[1*]

Material and methods

Mediterranean Diet Mix composition

The polyphenol profile of the mix of extracts was characterized by liquid chromatography coupled to tandem mass spectrometry (HPLC/MS/MS) analysis. Chromatographic separation was performed using an HPLC equipped with two micro-pumps Series 200 (Perkin Elmer, Norwalk, Connecticut, USA). MS/MS analyses were performed by an API 3000 triple quadruple mass spectrometer equipped with a TurboIonSpray source (Applied Biosystems, Foster City, California, USA).

Determination of urinary polyphenols

Urine from mice grouped in the same cage were pooled and 200 µL of urine sample were added to 800 µL of 0.2% acetic acid and after centrifugation at 16800xg for 5 min at 4°C the solution was loaded onto Oasis HLB (1 mL 30 mg sorbent) cartridges (Waters, USA). Polyphenols were eluted with 1mL of methanol 0.2% in acetic acid. The eluate was dried under a stream of nitrogen, and re-dissolved in 100 µL methanol/H_2O (70:30, v/v) immediately before the High Resolution Mass Spectrometry. Metabolites, including sulfated and glucuronidated compounds, were identified by comparison of their retention times and mass spectrometry data with those of reference compounds if available or assigned using exact mass values up to the fifth decimal digit with mass tolerance ± 5ppm.

Tables

Supplementary Table 1. Polyphenol content of the mix of extracts and contribution of each dietary component to the final composition

Dietary components	Polyphenols					
	Procyanidins	Phenolic acids	Flavonoids	Hydrolysable tannins	Phenyl ethyl alcohols	Secoiridoids
Apple (mM catechin eq)	0.16	0.52	0.32			
Tomato (mM naringenin eq)		0.58	0.42			
Walnuts (mM catechin eq)	0.26		0.02	0.72		
Whole wheat (mM ferulic acid eq)		1.00				
Olive oil (mM hydroxytirosol eq)		0.15			0.47	0.38
**Mix of extracts (mM tot eq)**	0.08	0.46	0.19	0.11	0.09	0.08

Supplementary Table 2. Composition of the animal diets

Component	HFWD		LFD
Casein (%)	24.5		24.5
Corn starch, pre-gelatinized (%)	7.8		30
Maltodextrin, 10 DE (%)	8		18
Sucrose (%)	28.319		11.949
Cellulose powder (%)	1		5
L-Cystine (%)	0.3		0.3
Vitamin (AIN mod.) premix (%)	1		1
Mineral & trace element premix (%)	4		4
Choline bitartrate (%)	0.25		0.25
TBHQ (%)	0.001		0.001
Dye (%)	0.03		optional
Pork lard (%)	22.6		0
Soybean oil (%)	2.2		5
Crude protein (%)	21.5		21.5
Crude fat (%)	24.9		5.2
Crude fiber (%)	1		5
Crude ash (%)	2.7		3.6
Starch (%)	7.5		28.9
Sucrose (%)	30.5		13.1
NfE (carbohydrates) (%)	46.9		60.7
Calcium (%)	0.06		0.64
Phosphorus (%)	0.42		0.43
Vitamin A (IU/Kg)	4		4
Vitamin D3 (IU/Kg)	125		1
Vitamin E (IU/Kg)	75		75
Fatty acids			
C 12:0 (%)	0.05		0.01
C 14:0 (%)	0.32		0.02
C 16:0 (%)	5.7		0.58
C 18:0 (%)	3.14		0.18
C 20:0 (%)	0.08		0.02
C 18:1 (%)	10.02		1.29
C 18:2 (%)	3.31		2.65
C 18:3 (%)	0.35		0.29
Physiological fuel value	20.8		15.7
Protein (Kcal%)	17		23
Fat (Kcal%)	45		12
Carbohydrates (Kcal%)	38		65

Supplementary Table 3. Molecular formula and selected ion for quantified polyphenols

Compound	Formula	M-H
Ferulic acid	$C_{10}H_{10}O_4$	193.05063
Dihydroferulic acid	$C_{10}H_{12}O_4$	195.06628
p-coumaric acid	$C_9H_8O_3$	163.04007
Dihydroferulic acid sulfate	$C_{10}H_{12}SO_7$	275
Dihydroferulic acid glucuronide	$C_{16}H_{20}O_{10}$	371.09837
Chlorogenic acid	$C_{16}H_{18}O_9$	353.08781
Caffeic acid glucoronide	$C_{15}H_{16}O_{10}$	355.06707
Caffeic acid sulfate	$C_9H_8SO_7$	258.9918
Dihydrocaffeic acid sulfate	$C_9H_{10}SO_7$	261.00745
Dihydrocaffeic acid glucuronide	$C_{15}H_{18}O_{10}$	357.08272
Protocatechuic acid	$C_7H_6O_4$	153.01933
Hippuric acid	$C_9H_9NO_3$	178.05097
Vanillic acid	$C_8H_8O_4$	167.03498
Caffeic acid	$C_9H_8O_4$	179.03498
Dihydrocaffeic acid (3,4-Dihydroxyphenylpropionic acid)	$C_9H_{10}O_4$	181.05063
di-HPA acid 3,4-Dihydroxyphenylacetic acid	$C_8H_8O_4$	167.03498
HPP acid 3-Hydroxyphenylpropionic acid	$C_9H_{10}O_3$	165.05572
Ferulic acid glucoronide	$C_{16}H_{18}O_{10}$	369.08272
Ferulic acid sulfate	$C_{10}H_{10}SO_7$	273.00745
3-HPA acid (3-Hydroxyphenylacetic acid)	$C_8H_8O_3$	151.04007
Homovanillic acid (3-Methoxy-4-hydroxyphenylacetic acid)	$C_9H_{10}O_4$	181.05063
4-Hydroxybenzoic acid	$C_7H_6O_3$	137.02442
3-Hydroxybenzoic acid	$C_7H_6O_3$	137.02442
4-Hydroxyhippuric acid	$C_9H_9NO_4$	194.04588
3-Hydroxyhippuric acid	$C_9H_9NO_4$	194.04588
Phenylacetic acid	$C_8H_8O_2$	135.04515
Furuloylglycine	$C_{12}H_{13}NO_5$	250.0721
Epicatechin	$C_{15}H_{14}O_6$	289.07176
Epicatechin glucuronide	$C_{21}H_{22}O_{12}$	465.10385
Epicatechin-O-sulfate	$C_{15}H_{14}O_9S$	369.02858
O-methyl-epicatechin-O-glucuronide	$C_{22}H_{24}O_{12}$	479.1195
O-methyl-epicatechin-O-sulfate	$C_{16}H_{16}O_9S$	383.04423
Syringic acid	$C_9H_{10}O_5$	197.04555
Uro-A glucuronide	$C_{19}H_{16}O_{10}$	403.06707
Uro-B-glucuronide	$C_{19}H_{16}O_9$	387.07216
Uro-C glucuronide	$C_{19}H_{16}O_{11}$	419.06198
Uro-C methyl ether glucuronide	$C_{20}H_{18}O_{11}$	433.07763
DMEA (dimethyl ellagic acid glucuronide)	$C_{22}H_{18}O_{14}$	505.06238
Uro-D glucuronide	$C_{19}H_{16}O_{12}$	435.0569
Uro-A	$C_{13}H_8O_4$	227.19332
Uro-B	$C_{13}H_8O_3$	211.04007
Uro-C	$C_{13}H_8O_5$	243.0299
Sinapinic acid	$C_{11}H_{12}O_5$	223.0612
Gallic acid	$C_7H_6O_5$	169.01425
Urolithin D	$C_{13}H_8O_6$	259.02481
Uro-A sulfate	$C_{13}H_8O_7S$	306.9918
Urolithin A sulfoglucuronide	$C_{19}H_{16}O_{13}S$	483.02388
Ellagic acid glucuronide	$C_{20}H_{14}O_{18}$	477.03108
Urolithin D methyl ether glucuronide	$C_{20}H_{18}O_{12}$	449.07255

Supplementary Table 4. Urinary concentration of polyphenols

Urinary polyphenols (ng/ml)	Mice arms					
	**Healthy LFD**	**Healthy HFWD**	**LFD**	**LFD-MD-MIX**	**HFWD**	**HFWD-MD-MIX**
Hippuric acid	2050	3028	3688	2861	4935	9512
4-Hydroxybenzoic acid	201	31	322	167	264	276
P-coumaric acid	0	0	5	4	34	16
M-coumaric acid	0	0	3	3	7	4
Chlorogenic acid	0	0	0	0	1	0
Dihydrocaffeic acid glucuronide	582	627	116	136	282	479
Ferulic acid glucoronide	1619	3707	1457	1440	5299	5922
Gallic acid	84	22	629	31	188	265
Siringic acid	918	1365	367	368	985	1213
Sinapinic acid	175	407	210	345	829	2481
Epicatechin	0	0	0	0	0	183
Hydroxyhippuric acid	2015	2138	1209	908	1803	2341
O-methyl-epicatechin-O-glucuronide	49	99	384	266	1047	278
Feruloylglycine	2218	1735	444	1222	1722	1228
**Total polyphenols**	9912	13160	8833	7751	17396	24196

Figures and figure legends

Supplementary Figure 1. Comparison of mice colon length at sacrifice (ANOVA p = 0.0056). Data are shown as individual values. n=5 Healthy LFD; n=4 Healthy HFWD; n=8 LFD; n=10 LFD-MD-MIX; n=6 HFWD; n=7 HFWD-MD-MIX. Statistical significance was obtained using one-way two-tailed ANOVA followed by Tukey's post-hoc test. * p < 0.05 vs. Healthy LFD; ** p < 0.01 vs. Healthy LFD.

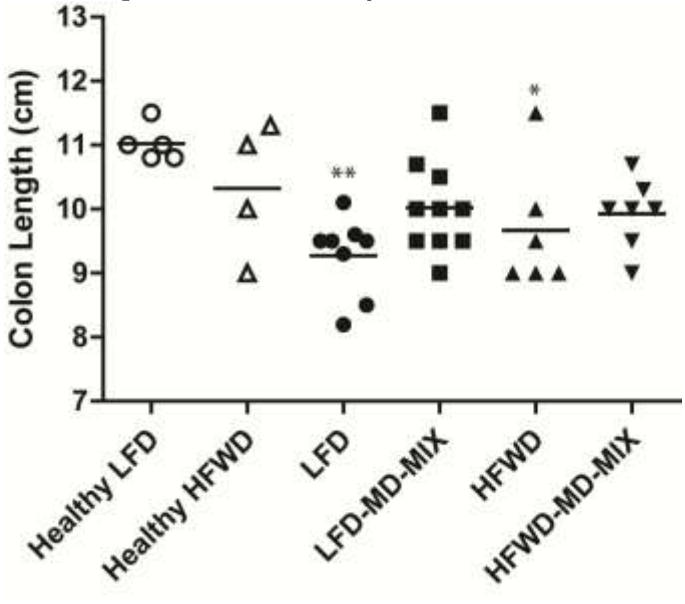

Supplementary Figure 2. Mice body weight at sacrifice (ANOVA p < 0.0001). Data are shown as individual values. n=5 Healthy LFD; n=4 Healthy HFWD; n=8 LFD; n=10 LFD-MD-MIX; n=6 HFWD; n=7 HFWD-MD-MIX. Statistical significance was obtained using one-way two-tailed ANOVA followed by Tukey's post-hoc test. * p < 0.05 vs. Healthy LFD; *** p < 0.001 vs. Healthy LFD; # p < 0.05 vs. Healthy HFWD; ## p <0.01 vs. Healthy HFWD.

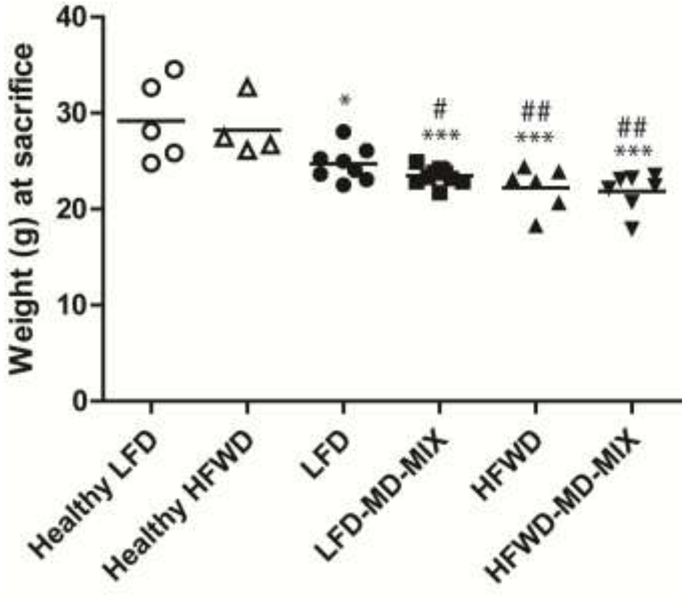

Supplementary Figure 3. Apoptosis analysis by q-PCR. Data are plotted as *Bax*/*Bcl2* ratio. Statistical analysis was performed using one-way two-tailed ANOVA followed by Tukey's post-hoc test. n=5 Healthy LFD; n=4 Healthy HFWD; n=8 LFD; n=10 LFD-MD-MIX; n=6 HFWD; n=7 HFWD-MD-MIX. # p < 0.05 vs. Healthy HFWD; ## p <0.01 vs. Healthy HFWD.

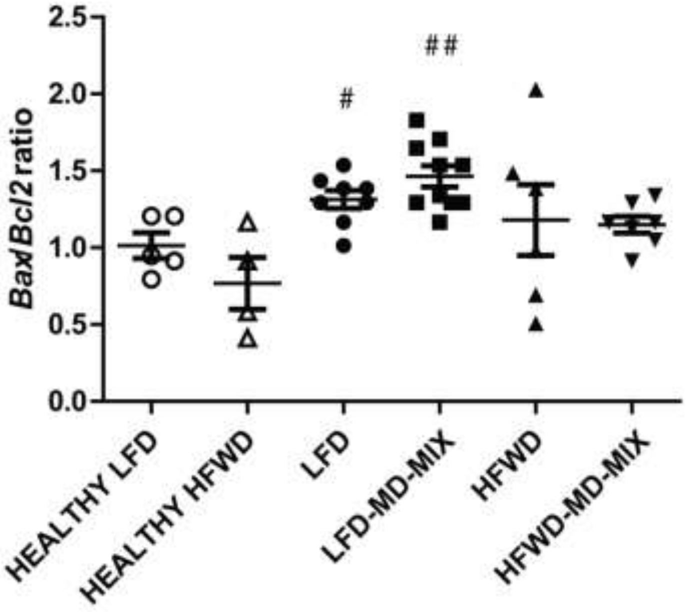

Supplementary Figure 4. Ki67 protein analyzed by IHC. Data are plotted as a percentage of Ki67 positive cells/total nuclei. Statistical analysis was performed using one-way two-tailed ANOVA. n=5 Healthy LFD; n=4 Healthy HFWD; n=8 LFD; n=10 LFD-MD-MIX; n=6 HFWD; n=7 HFWD-MD-MIX.

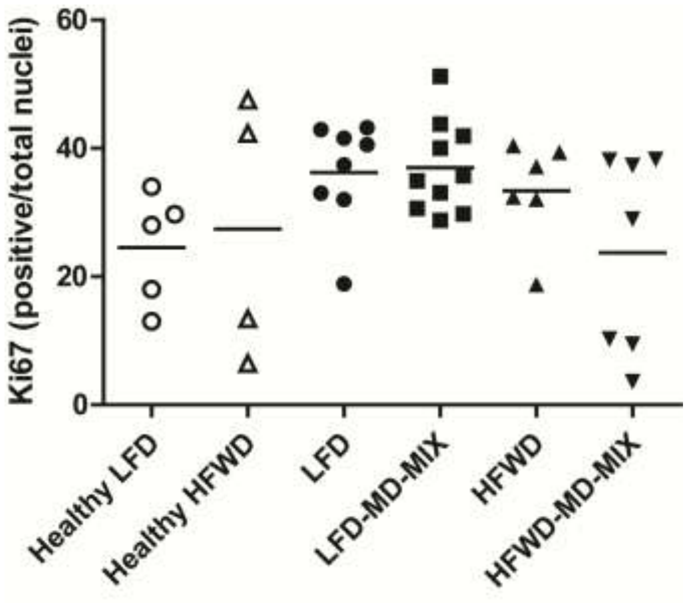

Supplementary Figure 5. PCoA based on weighted UniFrac distances among fecal microbiota samples of mice fed with LFD and HFWD diets, at time points T1 (filled circles), T2

(filled triangles) and T3 (filled squares). Healthy mice (upper panel), LFD and HFWD arms (middle panel), LFD-MD-MIX and HFWD-MD-MIX arms (lower panel). PCoA first (MDS1) and second (MDS2) principal components are plotted.

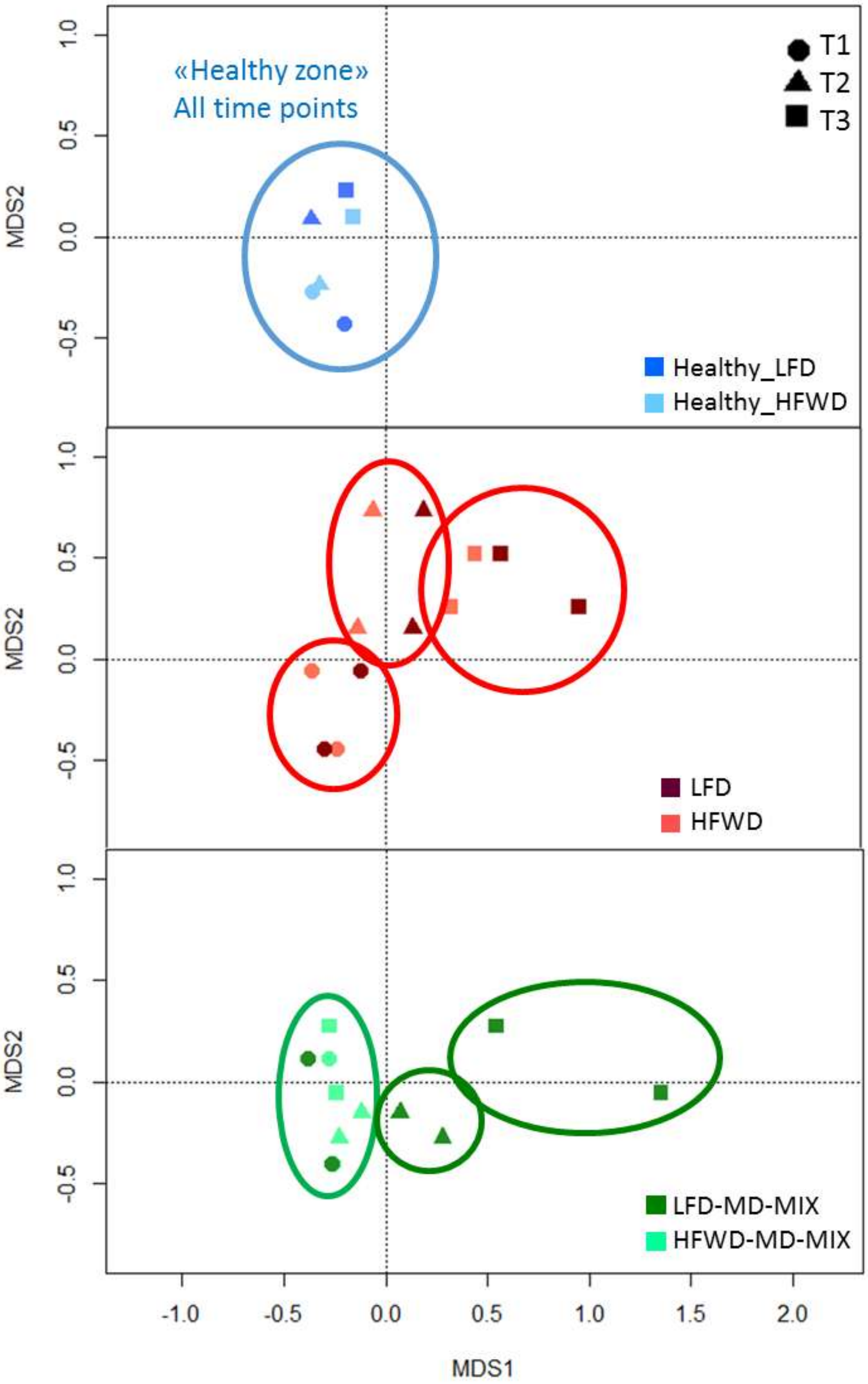

Supplement: Supplementary file 1 [file Data_Sheet_1.pdf]
